# Editorial: State of the art in immunopathologic mechanisms underlying preterm birth pathways and biomarkers for prematurity prediction

**DOI:** 10.3389/fped.2023.1163597

**Published:** 2023-03-24

**Authors:** Bruna Ribeiro de Andrade Ramos, Jossimara Polettini, Márcia Guimarães da Silva

**Affiliations:** ^1^ Department of Pathology, Botucatu Medical School, São Paulo State University (Unesp), Botucatu, Brazil; ^2^ University of Western São Paulo (Unoeste), Jaú, Brazil; ^3^ Federal University of Fronteira Sul, Passo fundo, Brazil

**Keywords:** preterm birth, immune response, biomarkers, adverse pregnancy outcomes, retinopathy, prematurity

**Editorial on the Research Topic** State of the art in immunopathologic mechanisms underlying preterm birth pathways and biomarkers for prematurity prediction

Preterm Birth (PTB) is a worldwide public health concern that accounts for 70% of neonatal morbidity and up to 20% of neonatal mortality (1, 2). The detrimental role of prematurity in short- and long-term sequelae is well known and includes cognitive impairment, neonatal complications, and increased susceptibility to infections. Multiple conditions lead to PTB—from maternal infections and environmental factors to hypertensive disorders of pregnancy (3). Despite many epidemiologic and pathophysiologic investigations, the complete understanding of mechanisms leading to spontaneous prematurity is yet to be achieved. The most triggered pathways include inflammation, senescence, and oxidative stress (4–6), nonetheless there are still no validated biomarkers to predict PTB (7, 8). In this context, integrated knowledge—from biochemic, microbiologic, and immunologic fields—is necessary to develop multivariable models to predict adverse outcomes (9, 10). This collection includes 5 articles covering the involvement of placental tissue, amniotic fluid, and maternal serum in deflagration of PTB and its complications; as well as investigations of inflammatory, behavioral, and genetic biomarkers of adverse pregnancy outcomes (APO).

As innate immune cells mediate the process of labor by releasing pro-inflammatory factors such as cytokines, chemokines and matrix metalloproteinases and adaptive immune cells play a pivotal role in the maintenance of maternal-fetal tolerance, a common feature shared by several APOs is the maternal-fetal immune disorder. Indeed, the crosstalk between tissues from the mother and the fetus may reflect the immune imbalance and initiate PTB pathways (11, 12). The role of several immune cell types has already been investigated in the maternal-fetal interface. Particularly, macrophages, which are important antigen-processing and presenting cells. The subtype M2 plays important roles in apoptotic cell clearance, tissue repair and remodeling, and a dysregulated polarization between subtypes M1 and M2 is associated with a variety of pregnancy complications (13). In the present Research Topic, Zha et al. reinforced that decidual M2 macrophages are decreased in the preterm labor group compared to term pregnancies, resulting in an increased M1/M2 ratio in this group. Therefore, the authors associated the breakdown of maternal-fetal immune tolerance during preterm birth with M1/M2 imbalance. Another essential role of macrophages in the setting of inflammatory syndromes, such as PTB, relies on their bidirectional communication with T cells, especially regulatory T cells (Treg). They present antigens to naive T cells, driving the differentiation to Treg, which limit the excessive inflammation. Indeed, recent evidence demonstrates a mechanistic role of decreased Treg in the pathophysiology of idiopathic preterm labor/birth and adverse neonatal outcomes. Understanding pathways involving immune cell regulation is essential to develop therapeutic targets to mitigate APOs (14, 15).

Immune response to intrauterine infection is a well-known trigger for PTB. Considering that 40% of preterm births are associated with infection and that neonatal adverse outcomes are linked to the exposure of the fetus to microorganisms (16), their detection and/or detection of inflammatory markers is an important field of research, as investigated by Matulova et al. The authors have conducted a retrospective study with preterm pregnancies and reported that the presence of intra-amniotic infection and inflammatory markers in the amniotic fluid was associated with an estimated fetal weight under the 10th and 25th percentiles.

Several neonatal complications are described in PTB cases, e.g., intracerebral hemorrhage, necrotizing enterocolitis, and retinopathy of prematurity (ROP) (17). ROP is a major cause of childhood blindness among infants born preterm. As this condition is frequently misdiagnosed (18), the identification of biomarkers can improve the diagnostic process as well as the assessment of disease prognosis. The original research provided by Li et al. revealed that altered expression of circular RNAs—stable non-coding RNAs—in the peripheral blood mononuclear cells may be used as potential biomarkers for treatment-requiring ROP infants.

The diverse immune response towards external factors is, to a great extent, the reflection of genetic features that are observed in some pregnancy complications that lead to PTB. Within this framework, Wang et al. have investigated genetic biomarkers, molecular mechanisms, and therapeutic strategies for preeclampsia (PE), a gestational hypertensive disorder directly linked to prematurity. Using the GSE75010 dataset they obtained differently expressed inflammation-related genes. Pathway analysis was performed, and they constructed machine-learning models that identified three key inflammation-associated genes, namely *INHBA*, *OPRK1*, and *TPBG*, which can be used as potential genetic biomarkers for preeclampsia prediction and treatment. The authors established a nomogram as a predictive model. Additionally, they provided insights into the mechanisms of preeclampsia development at the transcriptome level and performed corresponding drug predictions. Their contemporary approach is useful to predict treatment targets to be evaluated in future clinical studies.

Aside from infection, inflammation, and genetic factors, recent findings by Becker et al. highlight the importance of psychosocial characteristics and stress as risk factors for PTB. Considering the interconnectedness of APOs, the authors presented a refined work proposing a multitask machine learning approach to simultaneously evaluate and quantify stressors that contribute to APOs. It was demonstrated that life stress and perceived pregnancy risks were associated with gestational complications, which reinforces the negative impact of psychological factors on maternal and fetal health. Based on a single-cell immune dataset from a subset of patients, Becker and collaborators also linked stress-related factors to immune system properties, indicating that stress-related factors can predict components of the immune system. This study reinforces the need for an integrative immune and stress model of pregnancy to prevent multiple APOs at a time.

In summary, this Research topic provides the reader with insightful and updated information, while reinforcing the immune deviation of gestational tissues in PTB and the immediate deleterious consequences for the newborns (such as increased low birth weight rates). In this context, the works featured here show the value of the detection of biomarkers for such consequences (e.g., ROP) and the use of bioinformatic approaches to design prediction models for early detection of APOs (such as PE and PTB). Indeed, the use of machine learning models to address the multifactorial characteristics of APO will allow in the future a more integrated and personalized medicine towards the modification of environmental and behavioral factors related to them.
